# Potential Role and Impact of Peripheral Blood Mononuclear Cells in Radiographic Axial Spondyloarthritis-Associated Endothelial Dysfunction

**DOI:** 10.3390/diagnostics11061037

**Published:** 2021-06-04

**Authors:** Patricia Ruiz-Limon, Maria L. Ladehesa-Pineda, Clementina Lopez-Medina, Chary Lopez-Pedrera, Maria C. Abalos-Aguilera, Nuria Barbarroja, Isabel Arias-Quiros, Carlos Perez-Sanchez, Ivan Arias-de la Rosa, Rafaela Ortega-Castro, Alejandro Escudero-Contreras, Eduardo Collantes-Estevez, Yolanda Jimenez-Gomez

**Affiliations:** 1 Maimonides Biomedical Research Institute of Cordoba (IMIBIC), 14004 Córdoba, Spain; lourdesladehesapineda@gmail.com (M.L.L.-P.); clementinalopezmedina@gmail.com (C.L.-M.); rosario.lopez.exts@juntadeandalucia.es (C.L.-P.); mc.abalos@outlook.com (M.C.A.-A.); nuria.barbarroja.exts@juntadeandalucia.es (N.B.); isabel.arias.quiros@hotmail.com (I.A.-Q.); b32pesac@uco.es (C.P.-S.); ivan.arias@imibic.org (I.A.-d.l.R.); orcam84@hotmail.com (R.O.-C.); alexcudero@hotmail.com (A.E.-C.); educollantes@yahoo.es (E.C.-E.); 2 UGC Rheumatology, Reina Sofia University Hospital, 14004 Córdoba, Spain; 3 Department of Medicine (Medicine, Dermatology and Otorhinolaryngology), University of Córdoba, 14004 Córdoba, Spain; 4 UGC of Endocrinology and Nutrition, The Biomedical Research Institute of Málaga (IBIMA), Virgen de la Victoria Hospital, 29010 Málaga, Spain; 5 CIBER Physiopathology of Obesity and Nutrition (CIBEROBN), Carlos III Health Institute, 28029 Madrid, Spain

**Keywords:** endothelium, circulating mononuclear cells, inflammation, oxidative stress, radiographic axial spondyloarthritis

## Abstract

Endothelial dysfunction (ED) is well known as a process that can lead to atherosclerosis and is frequently presented in radiographic axial spondyloarthritis (r-axSpA) patients. Here, we investigated cellular and molecular mechanisms underlying r-axSpA-related ED, and analyzed the potential effect of peripheral blood mononuclear cells (PBMCs) in promoting endothelial injury in r-axSpA. A total of 30 r-axSpA patients and 32 healthy donors (HDs) were evaluated. The endothelial function, inflammatory and atherogenic profile, and oxidative stress were quantified. In vitro studies were designed to evaluate the effect of PBMCs from r-axSpA patients on aberrant endothelial activation. Compared to HDs, our study found that, associated with ED and the plasma proatherogenic profile present in r-axSpA, PBMCs from these patients displayed a pro-oxidative, proinflammatory, and proatherogenic phenotype, with most molecular changes noticed in lymphocytes. Correlation studies revealed the relationship between this phenotype and the microvascular function. Additional in vitro studies confirmed that PBMCs from r-axSpA patients promoted endothelial injury. Altogether, this study suggests the relevance of r-axSpA itself as a strong and independent cardiovascular risk factor, contributing to a dysfunctional endothelium and atherogenic status by aberrant activation of PBMCs. Lymphocytes could be the main contributors in the development of ED and subsequent atherosclerosis in this pathology.

## 1. Introduction

Radiographic axial spondyloarthritis (r-axSpA) is a chronic inflammatory disease that mainly affects the axial skeleton, frequently targeting the sacroiliac joints in the pelvis. Additional sites of involvement include the entheses and peripheral joints [1]. R-axSpA patients have about twofold greater risk of premature mortality than the general population, which is mainly caused by an augmented cardiovascular (CV) risk [2,3,4]. Among specific CV disorders, accelerated atherosclerosis possibly contributes to the increased CV risk observed in this pathology [5].

There are several factors in r-axSpA that may lead to the development of atherosclerosis and cardiovascular disease (CVD). Traditional risk factors such as hypertension and an atherogenic lipid profile [5,6] play a crucial role, but do not fully explain the substantial burden of atherosclerosis detected in this disorder [7]. Thus, it is likely that the generalized inflammatory state renders these patients more prone to develop atherosclerotic CVD, as inflammatory mechanisms associated with atherosclerosis pathogenesis resemble those observed in rheumatology diseases [8,9].

There exist multiple potential mechanisms whereby inflammation may contribute to atherosclerosis, and one of them is the development of a dysfunctional endothelium [10]. Endothelial dysfunction (ED) is a preceding step in the formation of structural atherosclerotic changes, and it has emerged as a useful predictor of long-term CV risk [11]. Its role in initiating the cascade of events leading to atherosclerosis makes this an early marker of disease at a point that may allow for adequate treatment strategies prior to the development of full-blown atherosclerosis [12].

A variety of studies have shown that ED is a vascular abnormality frequently presented in r-axSpA patients [13,14,15], and peripheral blood mononuclear cells (PBMCs) might play a key role in its triggering, initiation, and development. In r-axSpA, T lymphocytes, a cell subtype crucial in the immune system regulation [16], have been demonstrated to be central elements in the pathology [17]. The alterations of T cells found in r-axSpA patients include increased frequency of T helper (Th)-1, Th17, Th2, Th22, T cytotoxic (Tc)-1, and Tc17 cells [18,19,20], generating an skewness of a set of ratios such as Th1/Th2, Tc1/Tc2, Th17/regulatory T cells (Treg), and Tc1/Treg ratios as compared to the healthy population [19,21]. Th1 and Tc1 cells drive the cellular immunity, while Th2 and Tc2 cells play a critical role in humoral and atopic responses [22,23]. Other T-cell populations, including Th17 and Th22, are directly involved in and mediate chronic inflammatory response, and Treg cells suppress effector T-cell response [18,24]. The function of immune cells is achieved through the cytokine signature they can secrete; thus, an imbalanced proportion of T-cell subsets results in an alteration of expression levels of immune mediators involved in the pathogenesis and progression of diseases as r-axSpA [19,20]. In addition, blood B-cell subsets have been proven to have an essential role in r-axSpA pathogenesis, showing that a misbalance of these cells promotes the progression of r-axSpA [25]. On the other hands, r-axSpA-derived peripheral blood monocytes display an upregulation of proteins associated with inflammation, specifically those related to leukocyte extravasation, vascular endothelial growth factor (VEGF), Janus kinase/signal transducer and activator of transcription (JAK/STAT), and Toll-like receptor (TLR) signaling pathways, as well as proteins belonging to the ubiquitin proteasome pathway [26], thus demonstrating their implication in the r-axSpA pathogenesis. Nevertheless, the possible role of different circulating mononuclear cells in the development of ED has not yet been explored in r-axSpA.

In addition to inflammation, evidence suggests that oxidative status has a significant role in modulating endothelial function [27]. Plasma oxidative stress, a process induced via immune-mediated mechanisms, has been demonstrated to play a major role in the pathogenic process of r-axSpA [28,29,30,31], being associated with the inflammation, disease activity, and administered therapy in these patients [30,31]. Hence, oxidative stress produced by circulating PBMCs, actively participating in the inflammatory response, might contribute not only to r-axSpA but also to the development of ED and atherogenic status in this disorder.

Overall, despite an ever-growing list of results revealing a dysfunctional endothelium in r-axSpA patients, numerous gaps remain in our understanding of the precise mechanisms that control the ED in this pathology. Therefore, the purpose of this study was to investigate cellular and molecular mechanisms underlying r-axSpA-related ED, focusing on the atherogenic, inflammatory, and oxidative profile of circulating leukocyte subsets. We further evaluated the potential roles of PBMCs in promoting atherosclerosis-associated vessel wall damage in this inflammatory disease.

## 2. Materials and Methods

### 2.1. Study Design and Participants

Thirty r-axSpA patients and 32 healthy age- and gender-matched donors were included in this cross-sectional study after ethics committee approval was obtained at the Reina Sofía University Hospital (Córdoba, Spain). Eligible patients had to fulfill the Assessment of SpondyloArthritis international Society (ASAS) classification criteria for the classification of axSpA [32]. Patients who met any of the following criteria were excluded from participating in the study: pregnancy, malignancies, chronic infections, other rheumatology diseases, extra-articular manifestations (psoriasis, inflammatory bowel disease, uveitis), inability to understand the procedures to the protocol, and receiving biological therapy. The study was conducted according to the principles of the Declaration of Helsinki.

All participants enrolled provided written informed consent and underwent a comprehensive medical history, physical examination, and clinical chemistry analysis before enrolment. A case report form was used to collect the following clinical data: age, gender, disease duration, human leukocyte antigen (HLA)-B27 status, plasma cytokine levels (i.e., tumor necrosis factor-α (TNF-α; pg/mL) and interleukin-1β (IL-1β; pg/mL)), and current medications. Disease activity was evaluated by the Bath Ankylosing Spondylitis Disease Activity Index (BASDAI) [33], C-reactive protein (CRP; mg/L), and erythrocyte sedimentation rate (ESR; mm/h). Spinal mobility and functionality of r-axSpA patients was measured by the Bath Ankylosing Spondylitis Metrology Index (BASMI) and Bath Ankylosing Spondylitis Functionality Index (BASFI) [34,35]. Structural damage was assessed by the modified stoke Ankylosing Spondylitis Spine Score (mSASSS) [36]. Moreover, the following CV risk factors were collected: obesity (body mass index ≥30 kg/m^2^), type 2 diabetes mellitus (history of diabetes), hypertension (history of hypertension or antihypertensive therapy or blood pressure >140/90 mm Hg), smoking status (nonsmoker or smoker), and dyslipidemia (history of hypercholesterolemia or cholesterol-lowering therapy or an low-density lipoprotein (LDL) cholesterol above upper limit of normal). Atherogenic risk was calculated by atherogenic index (total cholesterol/high-density lipoprotein (HDL)) and established as >4.5 in female and >5 in male [37].

### 2.2. Blood Sample Collection and Assessment of Biological Parameters

Peripheral venous blood samples were collected and processed as previously indicated [38]. Plasma and serum were aliquoted and stored at −80 °C until their analysis. Inflammatory markers (ERS and CRP), genetic factors (HLA-B27), and lipid profile (total cholesterol, HDL cholesterol, LDL cholesterol, triglycerides, and apolipoproteins A and B) were quantified as part of routine patient management.

### 2.3. Endothelial Function

A Periflux System 5010 Laser-Doppler Flowmeter (Perimed, Järfalla, Stockholm, Sweden) was used to measure the microvascular function. The measurements were always taken by the same researcher, to avoid variability. With the patient sitting in a room with stable temperature (25–26 °C), the blood pressure cuff (Hokanson, Bellevue, WA, USA) was placed 5 cm above the elbow, while the receptor probe (only one probe was used) was placed on the forearm at 15 cm from the wrist (volar surface) of the same dominant hand. After a 5 min resting period, basal capillary flow was measured for 2 min. Thereafter, the cuff was inflated to suprasystolic pressure (180 to 220 mm Hg) and maintained in this way for 4 min. Subsequently, the cuff was rapidly deflated, and the flow was recorded for 3 min. The data obtained were recorded and automatically analyzed using the software PeriSoft for Windows. The software calculated several parameters, including the initial value, perfusion when occluded (Biological Zero), highest perfusion value after occlusion was released (Peak Flow), percentage change from the first to the maximum values, time to reach the maximum hyperemia, time to reach the half value after the maximum hyperemia, area of hyperemia, and rest flow.

### 2.4. Markers Related to Inflammation and Cellular Adhesion

For the analysis of inflammatory mediators (i.e., TNF-α, and IL-1β) in plasma samples, a Bio-Plex Pro Assay (Bio-Rad Laboratories, Hercules, CA, USA) was used according to the manufacturer´s instructions. The plasma concentrations of E-selectin, vascular cell adhesion molecule 1 (VCAM-1), and intercellular adhesion molecule 1 (ICAM-1) were examined using a Procarta Plex multiplex immunoassay (Thermo Fisher, Waltham, MA, USA), following the recommended protocol. Prepared plates were run on a Bio-Plex 200 System (BioRad Laboratories). Data were acquired and analyzed using the Bio-Plex Manager software version 6.0 (BioRad Laboratories). Plasma L-selectin was assessed by ELISA kits (BioNova, Madrid, Spain), according to the manufacturer´s protocol.

### 2.5. White-Blood-Cell Isolation

PBMCs from whole blood were separated by Ficoll gradient centrifugation (StemCell Technology, Oslo, Norway). Thereafter, the separation of monocytes and lymphocytes from the mononuclear layer was performed by the immunomagnetic depletion of non-monocytes, using commercially available kits (Monocyte Isolation kit II, Miltentyi Biotec Bergisch Gladbach, Germany). Purity of the fractions was assessed by flow cytometry (single-laser FACScalibur cytometer, BD Biosciences, San Jose, CA, USA), analyzing the size and complexity of each population (forward and size scatters). The purity was routinely ≥95% for both lymphocytes and monocytes.

### 2.6. Total RNA Isolation

Total RNA from leukocytes and human umbilical vein endothelial cells (HUVECs; Lonza Group Ltd., Basel, Switzerland) was extracted using TRIsure (Bioline, Taunton, MA, USA) following the manufacturer´s recommendations. A prerequisite for samples to be included in the RT-qPCR was a nondegraded pattern of the 18S and 28S subunits of ribosomal RNA in a 1.2% agarose gel. The RNA purity was verified by optical density absorption ratio OD260/OD280 that was between 1.8 and 2.0.

### 2.7. RT^2^ Profiler Atherosclerosis PCR Array

A human atherosclerosis RT^2^ Profiler PCR array (Qiagen, Hilden, Germany) was used to profile the expression of 84 genes related to atherosclerosis. The description of this PCR array can be found on the following website: http://www.sabiosciences.com/rt_pcr_product/HTML/PAHS-038Z.html (1 January 2016). Specifically, single-stranded cDNA was synthesized from 500 ng of total RNA from two samples (each consisted of a pool of three RNA samples of PBMCs from r-axSpA patients or healthy donors (HDs)), using an RT^2^ first-strand cDNA synthesis kit (Qiagen). Next, cDNAs were mixed with RT^2^ SYBR Green qPCR Mastermix (Qiagen), and real-time PCR was performed according to the manufacturer’s protocol. Quantitative real-time PCR was performed on a LightCycler^®^ Thermal Cycler System (Roche Diagnostics, Indianapolis, IN, USA). Four housekeeping genes (i.e., β-actin, b2-microglobulin, hypoxanthine phosphoribosyl transferase 1, and ribosomal protein large p1) were used for normalization. The gene expression values were calculated using the 2^−ΔΔCt^ method. All measurements were performed in duplicate.

### 2.8. RT-qPCR Analysis

RT-qPCR was conducted in two steps. For the first-strand cDNA synthesis, 1 µg of total RNA was reverse-transcribed using random hexamers as primers and Transcriptor Reverse Transcriptase (QuantiTect^®^ Reverse Transcription kit (Qiagen)). Gene expression was assessed by real-time PCR using a LightCycler^®^ Thermal Cycler System (Roche Diagnostics). The reaction was carried out according to manufacturer’s protocol. The primers are listed in Table S1 (Supplementary Materials). The reactions consisted of an initial denaturing of 10 min at 95 °C, then 40 cycles of a 15 s denaturing phase at 95 °C, and a 1 min annealing and extension phase at 60 °C. The amplification curves were analyzed using the Roche LC software, both for determination of Ct and for melting curve analysis. Fidelity of the PCR was determined by melting temperature analysis. The gene expression values were calculated using the 2^−ΔΔCt^ method and normalized to the mean of glyceraldehyde-3-phosphate dehydrogenase (GAPDH). All measurements were performed in duplicate. Controls consisting of reaction mixture without cDNA were negative in all runs.

### 2.9. Determination of Plasma Oxidative Stress Biomarkers

The nitric oxide (NO) stable end products nitrite plus nitrate were measured in plasma using a Total Nitric Oxide Assay kit (Thermo Scientific, Rockford, IL, USA). Serum total antioxidant capacity (TAC) was determined by quantitative colorimetric determination, using a TAC Assay Kit (BioVision, Mountain View, CA, USA). The plasma lipoperoxydes (LPO) were quantified using a Lipid Hydroperoxide assay kit (Cayman Chemical, Ann Arbor, MI, USA).

### 2.10. Determination of Oxidative Stress Biomarkers in White Blood Cells

Oxidative stress biomarkers were analyzed in lymphocytes and monocytes using the single-laser FACSCalibur (BD Biosciences) cytometer. Test standardization and data acquisition analysis were performed using the CELL Quest software (BD Biosciences). A forward and side scatter gate was used for the selection and analysis of the different cell subpopulations.

For the assessment of reactive oxygen species (ROS) generation, including peroxides and peroxynitrites, cells were incubated with 20.5 µM dichloro-dihydro-fluorescein diacetate (DCFHDA; Sigma-Aldrich, St Louis, MI, USA) and 5 µM dihydrorhodamine-123 (DHRH-123; Sigma-Aldrich) for 30 min in the dark at 37 °C. For the detection of intracellular glutathione (GSH), cells were incubated with 1 µM 5-chloromethylfluorescein diacetate (CMF-DA; Invitrogen, Eugene, OR, USA) for 30 min in the dark at 37 °C. Then, cells were washed, resuspended in PBS, and analyzed on a single-laser FACSCalibur (BD Biosciences) cytometer. The JC1 Mitoscreen assay (BD Biosciences) was used (final concentration 2 µM) to assess mitochondrial membrane potential (ΔΨm) according to the manufacturer’s instructions.

Mitochondrial superoxide dismutase (SOD2), catalase, and glutathione peroxidase (GPx) activities were assayed in cell lysates using specific kits (Cayman Chemical) following the manufacturer’s recommendations.

### 2.11. Protein Extraction and Western Blotting

Nuclear extracts were prepared according to standard protocols [39], and the Bradford assay method (Bio-Rad Laboratories) was used to determine protein concentration. Then, 10 µg of nuclear protein per sample was separated by SDS-PAGE using 4–20% gradient gels (Bio-Rad) and transferred to nitrocellulose membranes (Bio-Rad). After Ponceau staining to ensure equal sample loading, membranes were blocked for 1 h with 3% BSA in TBS buffer (20 mM Tris pH 7.4, 150 mM NaCl) with 0.05% Tween-20. After overnight incubation at 4 °C with anti-human nuclear factor-κB (NF-κB) antibody (1:500) (Santa Cruz Biotechnology, Inc., Santa Cruz, CA, USA), membranes were incubated with the appropriate IgG HRP-conjugated secondary antibody (1:7500) (Santa Cruz Biotechnology). Immunoreactive bands were visualized with an enhanced chemiluminescence reagent (Lumigen ECL Ultra; Lumigen Inc. Southfield, MI, USA) using an ImageQuant^TM^ L4000 biomolecular imager (GE healthcare BioScience AB, Uppsala, Sweden). Band intensities were quantified using Image J software followed by normalization to Ponceau staining [40].

### 2.12. In Vitro Studies

Endothelial cells (HUVECs; Lonza Group Ltd.) were cultured in Endothelial Cell Basal medium (EBM; Lonza Group Ltd.) supplemented with an EBM-bullet kit (Lonza Group Ltd.) and 10% FBS (BioWest, Nuaillé, France) at 37 °C in a humidified 5% CO_2_ atmosphere. For in vitro studies, HUVECs were seeded into six-well plates (4 × 10^5^ cells per well). All the experiments were performed after reaching 100% confluence.

For coculture experiments, PBMCs were isolated from r-axSpA patients (*n* = 5) and HDs (*n* = 5) as previously described in this section. Then, PBMCs were seeded into Transwell inserts (Corning^®^ Transwell^®^ polycarbonate membrane cell culture inserts, Sigma-Aldrich) (1 × 10^6^ cells per Transwell) in EBM, before adding them into multiple-plate wells preloaded with HUVECs for 24 h. At the end of the experiments, HUVECs were harvested for RNA and protein determinations or processed for flow cytometry.

### 2.13. Statistical Analysis

Statistical analysis used SPSS statistical software, version 19.0 for WINDOWS (SPSS Inc., Chicago, IL, USA). All data in text, figures, and tables are expressed as mean ± SD. The normal distribution of variables (Gaussian data) was assessed using the Kolmogorov–Smirnov test. For continuous data, comparison among variables were performed using Student’s tests for Gaussian data and using Mann–Whitney rank sum tests for non-normally distributed data. Categorical data were analyzed using the *χ*^2^ test or Fisher’s exact probability test, as appropriate. Correlations were assessed by Spearman´s rank correlation. A *p*-value <0.05 was considered statistically significant.

## 3. Results

### 3.1. Study Population

Clinical and laboratory parameters of the r-axSpA patients and HDs included in the study are summary in Table 1.

Patients showed nonsignificant difference in the frequency of obesity, type 2 diabetes, dyslipidemia, hypertension, smoking, and atherogenic risk as compared to HDs. Notwithstanding, the analysis of microvascular reactivity demonstrated that r-axSpA patients displayed ED, as shown by a substantial decrease in the rest flow (RF), the highest perfusion value after occlusion was released (Peak Flow, PF), the area of hyperemia (AH), the PF − RF, and the biological zero (BZ)-PF, as compared to HDs (*p* < 0.05; a representative image is shown in Figure S1, Supplementary Materials). In addition, the lipid profile analysis revealed lower concentrations of HDL in r-axSpA patients respect to HDs (*p* = 0.014), although those were within physiological baseline levels. There were no significant differences between r-axSpA patients and HDs in the remaining lipid profile parameters.

### 3.2. Identification of Proatherogenic Expression Profile in Circulating Leukocytes from r-axSpA Patients

**Figure 1:**
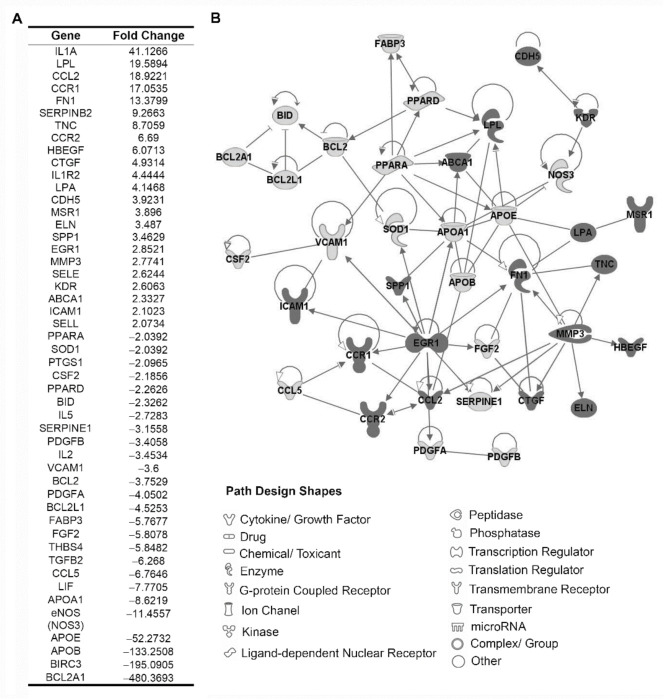
Atherogenic expression profile in PBMCs using atherosclerosis RT2 Profiler PCR array. (**A**) Fold change of 49 differentially expressed genes (≥2) between PBMCs from r-axSpA patients and HDs after normalization of the raw data. (**B**) Gene network between the modulated genes in r-axSpA patients. PBMCs, peripheral blood mononuclear cells; HDs, healthy donors; r-axSpA, radiographic axial spondyloarthritis.

To evaluate the expression profile of genes associated to atherogenic process in PBMCs from r-axSpA patients and HDs, a total of 84 genes were analyzed in two sets of RNA samples (each of them consisted of a pool of three RNA samples of PBMCs from r-axSpA patients and HDs), using a PCR array (Figure 1). After normalization of the raw data, the expression levels of 23 genes were found elevated, whereas 26 genes were discovered lower in r-axSpA patients compared with HDs (fold change ≥2; Figure 1A). Functional analysis showed that these genes were involved, among others, in inflammation, oxidative status, cellular adhesion, and metabolism/lipid transport. A gene network showing the functional inter-relationship between the modulated genes in r-axSpA patients (performed using the Ingenuity Pathways Analysis software) is displayed in Figure 1B.

**Figure 2:**
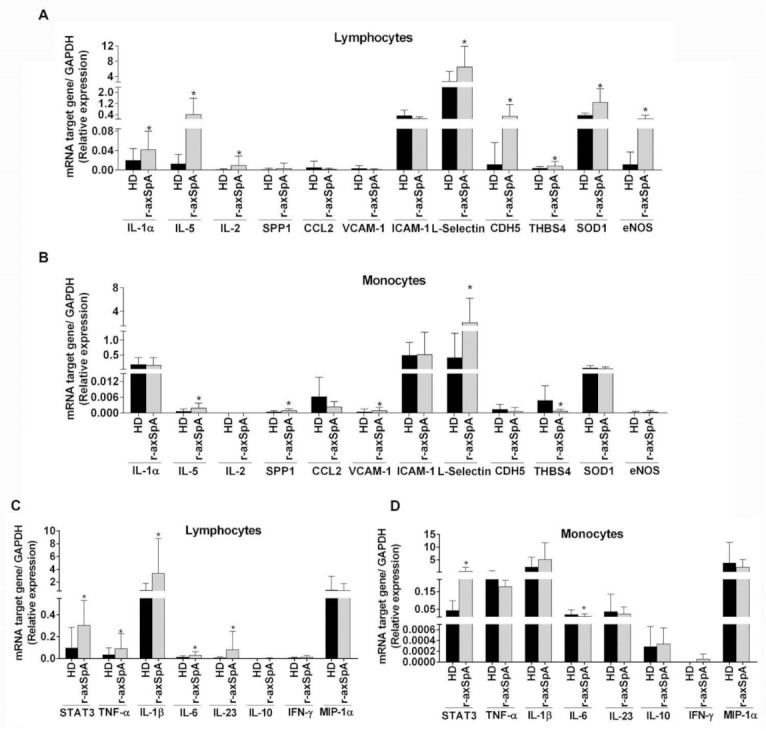
Study of atherogenic and inflammatory expression profile in lymphocytes and monocytes from r-axSpA patients. (**A**,**B**) Relative expression levels of atherogenic genes in lymphocytes (**A**) and monocytes (**B**) (HDs, *n* = 32; r-axSpA patients, *n* = 30). (**C**,**D**) Relative expression levels of inflammatory genes in lymphocytes (**C**) and monocytes (**D**) (HDs, *n* = 32; r-axSpA patients, *n* = 30). (**A**–**D**) Values are presented as means ± SD. The data were analyzed using an independent samples *t*-test or a Mann–Whitney U test. * vs. HDs (*p* < 0.05). CCL, C–C motif chemokine ligand; CDH, cadherin; eNOS, endothelial nitric oxide synthase; HDs, healthy donors; ICAM, intercellular adhesion molecule; IFN, interferon; IL, interleukin; MIP, macrophage inflammatory protein; r-axSpA, radiographic axial spondyloarthritis; SOD, superoxide dismutase; SPP, secreted phosphoprotein; STAT, signal transducer and activator of transcription; THBS, trombospondin; TNF, tumor necrosis factor; VCAM, vascular cell adhesion molecule.

Subsequently, we selected 12 differentially expressed genes on the basis of their expression levels (fold-change ≥2) and relevance in the atherogenesis process for further determination in circulating lymphocytes and monocytes from all the subjects recruited to the study (r-axSpA patients (*n* = 30) and HDs (*n* = 32)). As shown in Figure 2A, eight of 12 determined genes were found increased in lymphocytes from r-axSpA patients vs. HDs. Specifically, r-axSpA patients showed higher expression levels of genes associated with inflammatory/oxidative response (i.e., mRNA for IL-1α, IL-5, IL-2, endothelial nitric oxide synthase (eNOS), and superoxide dismutase (SOD)-1) and cellular adhesion (i.e., mRNA for L-selectin, cadherin 5 (CDH5), and trombospondin 4 (THBS4)) (*p* < 0.05). Concerning monocytes, higher expression of markers related to inflammatory response (i.e., mRNA for IL-5, secreted phosphoprotein 1 (SPP1)) and cellular adhesion (i.e., mRNA for VCAM-1, L-selectin), as well as lower expression levels for mRNA THBS4, was exhibited in r-axSpA patients vs. HDs (*p* < 0.05; Figure 2B). Nonsignificant changes between patients and controls were observed in the remaining studied parameters in immune cells (Figure 2A,B).

### 3.3. Plasma Atherogenic Markers in r-axSpA Patients

Given the aberrant expression of atherogenic markers in circulating leukocytes from r-axSpA patients, we next explored plasma parameters closely related to ED and atherosclerotic process (Figure S2, Supplementary Materials). The analysis of cellular adhesion proteins demonstrated higher plasma concentrations of L-selectin (*p* = 0.033), E-selectin (*p* = 0.036), and VCAM-1 (*p* = 0.037) in r-axSpA patients vs. HDs. There was no significant difference in plasma ICAM-1 levels when comparing both study populations (Figure S2, Supplementary Materials).

### 3.4. Analysis of Inflammatory Expression Profile in Circulating Leukocytes from r-axSpA Patients

Chronic inflammation promoted by immune system cells may be the major risk factor for atherosclerosis in r-axSpA [3]. In this regard, we next quantified the expression levels of several inflammatory pathway components in circulating leukocyte subsets (lymphocytes and monocytes). We found an increase in gene expression for STAT3, TNF-α, IL-1β, IL-6, and IL-23 (*p* < 0.05) in lymphocytes from r-axSpA patients as compared to HDs (Figure 2C). In addition, we observed that monocytes from r-axSpA patients displayed an alteration in mRNA levels for STAT3, IL-6, and interferon (IFN)-γ (Figure 2D) with respect to HDs. Nonsignificant differences between patients and controls were observed in the remaining analyzed variables in the different leukocyte subsets.

### 3.5. Oxidative Status in Plasma and Circulating Leukocyte Subsets from r-axSpA Patients

**Figure 3:**
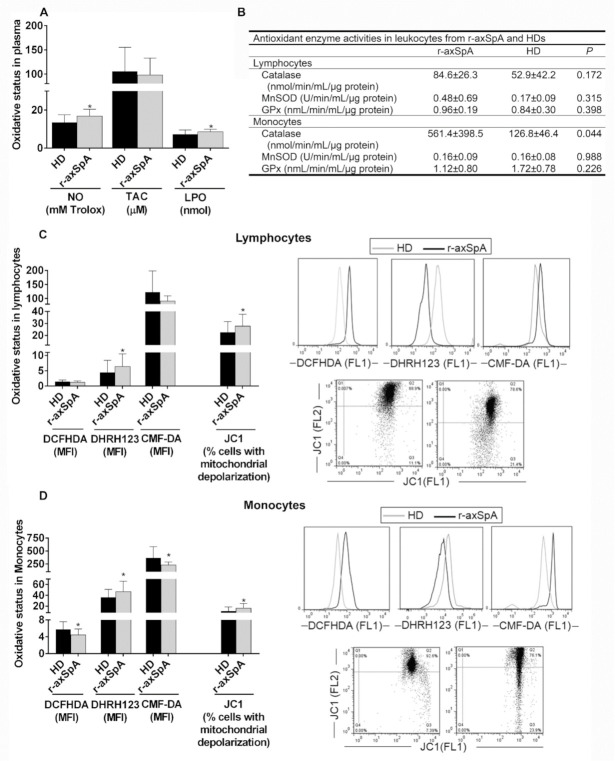
Oxidative status analysis in plasma and circulating mononuclear cells from r-axSpA patients. (**A**) Circulating levels of nitric oxide (NO), total antioxidant capacity (TAC), and LPO. (**B**) Catalase, mitochondrial superoxide dismutase (MnSOD), and glutathione peroxidase (GPx) in leukocytes from r-axSpA patients, and HDs. (**C**,**D**) Peroxide and peroxynitrite production, intracellular glutathione (GSH) levels, and mitochondrial membrane potential in lymphocytes (**C**) and monocytes (**D**). Representative histograms and dot plots of an r-axSpA patient and HDs are shown in right panels. (**A**–**D**) Values are presented as means ± SD (HDs, *n* = 32; r-axSpA patients, *n* = 30). The data were analyzed using an independent samples *t*-test or a Mann–Whitney U test. * vs. HDs (*p* < 0.05). HDs, healthy donors; r-axSpA, radiographic axial spondyloarthritis.

As previously described by our group [30,31], plasma oxidative status was higher in r-axSpA patients than HDs, as demonstrated by elevated plasma levels of NO and LPO present in this disorder (Figure 3A). Accordingly, augmented oxidative stress was observed on circulating leukocyte subsets (Figure 3B–D). Indeed, the peroxynitrite levels and percentage of cells with mitochondrial depolarization were upregulated in lymphocytes from r-axSpA patients vs. HDs (*p* < 0.05; Figure 3C). The augmented peroxynitrites levels in this cell subtype might be at least in part attributed to an elevated expression and an uncoupling of eNOS, a fact previously observed under pathological conditions that contribute significantly to ED [41]. In addition, we found the total GSH and peroxides levels to be reduced, whilst the peroxynitrite levels, percentage of cells with mitochondrial depolarization, and catalase activity were upregulated in monocytes (*p* < 0.05; Figure 3B,D) from r-axSpA patients as compared to HDs.

### 3.6. Correlation Studies between Atherogenic Status and Endothelial Function

Correlation studies demonstrated that the expression levels of several atherogenic, inflammatory, and oxidative mediators in mononuclear cells and plasma were inversely related to the microvascular function, pointing at a proatherogenic profile in r-axSpA (Table S2, Supplementary Materials). Specifically, mRNA expression levels of lymphocyte CDH5, eNOS, and inflammatory markers (STAT3, IL-1α, IL-1β, TNF-α, and IL-23), as well as monocyte inflammatory parameters (STAT3 and SPP1), were moderately associated with ED markers. In addition, plasma concentrations of cytokines (TNF-α and IL-1β) and cellular adhesion molecules (E-selectin and VCAM-1) were moderately and strongly correlated to ED parameters (Table S2, Supplementary Materials).

### 3.7. In Vitro Study of the Effect of PBMCs from r-axSpA Patients on Endothelium Activation

**Figure 4:**
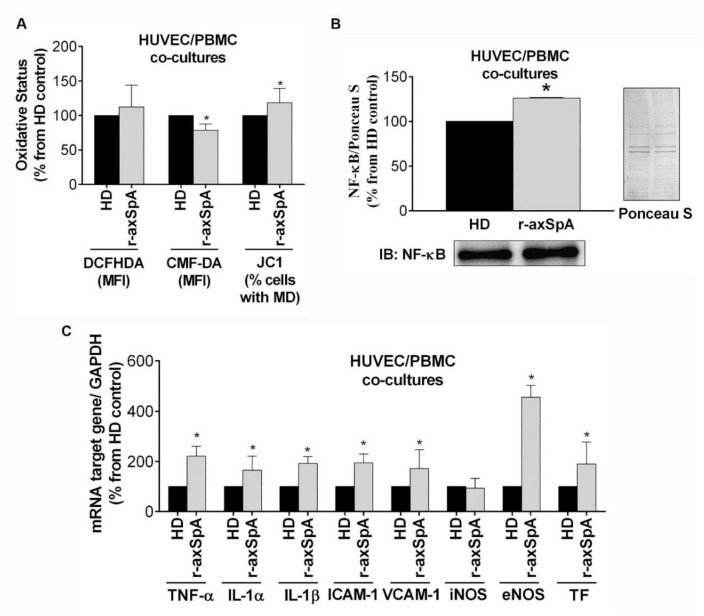
Analysis of the effect of PBMCs from r-axSpA patients on endothelial activation. (**A**–**C**) Peripheral blood mononuclear cells (PBMCs) isolated from r-axSpA patients (*n* = 5) and HDs (*n* = 5) were cocultured with human umbilical vein endothelial cells (HUVECs) for 24 h. (**A**) Peroxide production, intracellular glutathione (GSH) levels, and mitochondrial membrane potential in HUVECs. (**B**) Nuclear factor-κB (NF-κB) protein expression in HUVECs. (**C)** Expression levels of a panel of NF-κB target genes in HUVECs. Values are presented as means ± SD. The data were analyzed using an independent samples *t*-test or a Mann–Whitney U test. * vs. HDs (*p* < 0.05). eNOS, endothelial nitric oxide synthase; HDs, healthy donors; ICAM, intercellular adhesion molecule; IL, interleukin; iNOS, inducible nitric oxide synthase; r-axSpA, radiographic axial spondyloarthritis; TNF, tumor necrosis factor; TF, tissue factor; VCAM, vascular cell adhesion molecule.

On the basis of our in vivo data and given the role of mononuclear cells as key players in r-axSpA pathogenesis, we next investigated in vitro potential changes induced by r-axSpA PBMCs in endothelial activation-associated molecules. Our data showed the role of PBMCs in promoting impaired endothelial function in r-axSpA. Indeed, the results indicated a decrease in total GSH levels and increase in percentage of cells with mitochondrial depolarization (*p* < 0.050) (Figure 4A) after 24 h treatment of HUVECs with PBMCs from r-axSpA patients as compared to those from HDs. This increase in oxidative status by the r-axSpA PBMCs was associated with an increase in nuclear levels of NF-κB (*p* = 0.005; Figure 4B), as well as higher NF-κB target gene expression (mRNA for TNF-α, IL-1α, IL-1β, ICAM-1, VCAM-1, eNOS, and tissue factor (TF)) when HUVECs were cocultured with r-axSpA PBMCs vs. HD PBMCs (*p* < 0.050; Figure 4C).

## 4. Discussion

To best of our knowledge, this is the first study to exhaustively explore the molecular profiles of PBMCs (i.e., lymphocytes and monocytes) associated with the proatherogenic status of r-axSpA patients, as well as their potential role in mediating endothelial injury in this disorder. Our data indicate that PBMCs from r-axSpA patients display a proatherogenic profile, demonstrated by an altered expression of inflammatory markers, a higher expression of migratory factors, and an augmented oxidative burden, with most molecular changes noticed in lymphocytes. Moreover, our study shows that this phenotype in r-axSpA patients is associated with endothelial dysfunction. In vitro study confirmed the aberrant endothelial activation in response to PBMCs from r-axSpA patients.

Radiographic axial SpA is a pathology related to accelerated atherosclerosis and augmented CV morbidity/mortality [42]. An initial step in the atherosclerotic process is the aberrant activation of the endothelium, a cell layer which serves as the interface for multiple converging CV risk factors. While it is clear that r-axSpA patients have impaired endothelial function [14], an alteration also observed in our present study, the underlying role of r-axSpA immune system cells in ED and subsequent atherosclerosis is not completely understood in this pathology.

Over the last few years, several research groups have demonstrated that systemic inflammation plays a crucial role in promoting ED and accelerated atherosclerosis [10], thereby supporting the idea that chronic systemic inflammation may be a main contributor to the development of a dysfunctional endothelium in r-axSpA. In our study, we found that plasma inflammatory status was associated with endothelial function, a fact previously observed by van Eijk et al. [14] and Batko et al. [15], who showed that disease activity was related to microvascular dysfunction and that a reduction in inflammation with TNF-α blockade improved ED in r-axSpA patients. Notwithstanding, the cell determinants of such r-axSpA-associated endothelial abnormalities are unknown. This information could prove useful for development of risk stratification and intervention strategies in this pathology.

Our data demonstrated an inflammatory and migratory phenotype of r-axSpA lymphocytes, displayed by an augmented mRNA expression of markers for inflammation (i.e., TNF-α, IL-1α, IL-1β, IL-2, IL-6, IL-23, and IL-5) and adhesion (i.e., L-selectin, CDH5, and THBS4) as compared to healthy controls, a fact associated with the proinflammatory and proatherogenic profile observed in this pathology.

TNF-α is a good marker of subclinical atherosclerosis in r-axSpA [43], inasmuch as it favors not only the expression of IL-1β, monocyte chemoattractant protein (MCP)-1, ICAM-1, and VCAM-1, molecules involved in the leukocyte recruitment and the perpetuation of the inflammation and atherogenesis, but also the accumulation of apoptotic cells [44,45]. IL-1β induces cytokine production and, along with IL-6, promotes the extravasation of leukocytes into the atherosclerotic plaque [44]. In addition, elevated expression levels of proinflammatory cytokines IL-1α, IL-6, IL-2, and IL-23 were revealed in lymphocytes from our cohort of patients vs. health control subjects. These cytokines have been previously identified to be elevated at the circulating level in r-axSpA patients [46,47,48], and all of them have been demonstrated to be essential for the development of atherogenesis [49,50,51]. Notably, IL-5 expression levels, an anti-inflammatory cytokine, were also higher in lymphocytes from r-axSpA patients. This cytokine is mainly expressed by Th2 lymphocytes in atherosclerotic lesions [52], evidencing a possible crucial mechanism to counter the proinflammatory cytokine production in the development of atherogenic plaque.

Adhesion molecules are related to an increased risk for CVD. To date, there exist limited studies regarding the association of adhesion molecules and r-axSpA, and even contradictory information is currently available on this area. A few data have pointed to elevated circulating levels of ICAM-1 [53], whereas others have found nonsignificant alterations in circulating ICAM-1, VCAM-1, E-selectin, or L-selectin in r-axSpA [54,55]. In our study, we observed high levels of soluble L-selectin, E-selectin, and VCAM-1, along with an increased expression of adhesion molecules L-selectin, CDH5, and THBS4 at the mRNA level on lymphocytes from r-axSpA patients as compared to those from healthy controls, indicating the role of this leukocyte subset in migration and adhesion in r-axSpA.

Concerning monocytes from r-axSpA patients, this study demonstrated that this mononuclear cell also displayed a proatherogenic profile, showing an altered expression of inflammatory cytokines and factors for migration and adhesion. Indeed, we reported higher mRNA expression levels for IFN-γ and osteopontin (OPN, a cytokine codified by *SPP1* gene) on monocytes from r-axSpA patients vs. HDs. Both IFN-γ and OPN are inflammatory molecules associated with the atherogenic process [44,56] that were previously shown to be significantly increased at the circulating level in r-axSpA patients when compared to healthy control [48,57]. In addition, Choi et al. [57] demonstrated higher mRNA expression for OPN in PBMCs from r-axSpA vs. healthy control. This cytokine regulates, at least partially, the INF-γ expression in monocytes/macrophages and is associated with their recruitment and activation at lesion sites by regulating monocyte adhesion, migration, and differentiation, as well as inflammatory gene expression [58,59]. In contrast, expression levels for IL-6 were reduced, a fact previously observed in diabetic patients with atherosclerotic complications [60]. In addition, mRNA expression levels for IL-5, and adhesion molecules L-selectin and VCAM-1 were elevated, whilst a reduction in THBS-4 mRNA was also detected in r-axSpA monocyte as compared to HDs.

The cellular responses observed in PBMCs, related to the atherogenic process, depend on the activation of specific signaling pathways. In particular, we found an increased expression of transcriptional factor STAT3 at the mRNA level in r-axSpA vs. HDs, a transcription factor associated with the atherogenic process [61]. High-level expression of this factor is sufficient for leading the transcription of genes in the presence or absence of tyrosine phosphorylation [62], the latter via its ability to interact with unphosphorylated NF-κB [63]. This interaction would regulate the binding of this complex to the κB elements of the promoter to induce their transcription [63].

Alteration in oxidative status is also closely connected to ED. Our group previously reported an altered oxidative status in plasma from r-axSpA patients [30,31]. The present work extends these results, showing an increased plasma oxidative stress in r-axSpA, demonstrated by an increase in LPO and NO concentrations. Likewise, compared to HDs, our data provide, for the first time, evidence of elevated oxidative stress on mononuclear cells from r-axSpA patients that in turn may influence the expression of proatherogenic and proinflammatory molecules. In particular, r-axSpA lymphocytes and monocytes had an oxidant–antioxidant imbalance and significant losses of ∆ψm, revealing a large proportion of immune system cells containing mitochondria that lost the capacity to function optimally.

Overall, although further studies are required, our results reinforce what has been described in the literature in other rheumatology pathologies, that is, the unquestionable atherogenic profile of mononuclear cells in r-axSpA, underlining lymphocytes as the main contributor in this pathology. Therefore, the aberrant activation of PBMCs may be essentials in the development of ED and subsequent atherosclerosis in r-axSpA. Certainly, our correlation studies confirmed the relationship between the proatherogenic, proinflammatory and pro-oxidative profile present in circulating mononuclear cells and plasma and the ED observed in this pathology. In addition, our in vitro studies supported the implication of PBMCs from r-axSpA patients in promoting endothelial injury, the early stage in the formation of structural atherosclerotic changes, shown by elevated oxidative stress and an increase in inflammatory and atherogenic markers after coculturing endothelial cells with r-axSpA PBMCs as compared to those from HDs.

Our overall findings in this cross-sectional study suggest that r-axSpA may be a strong and independent CV risk factor, contributing to ED and, consequently, atherogenesis via aberrant activation of the immune system, with lymphocytes being the main white blood cells associated with the atherogenic profile observed in this pathology. Preventing premature CVD remains an important challenge in the management of r-axSpA patients, and our data could be useful for the development of more aggressive prophylaxis in this population before the development of full-blown atherosclerosis.

## Figures and Tables

**Table 1:** Clinical and laboratory parameters of the radiographic axial spondyloarthritis patients and the healthy donors.

	r-axSpA Patients (*n* = 30)	Healthy Donors (*n* = 32)	*p*
Clinical parameters			
Women/men, *n*/*n*	5/25	10/22	0.180
Age, years	46.40 ± 13.41	41.06 ± 10.04	0.080
BASDAI	4.42 ± 2.49	…	
BASFI	4.73 ± 3.32	…	
BASMI	3.22 ± 1.68	…	
mSASSS	19.16 ± 21.14	…	
Disease duration, years	13.23 ± 11.33	…	
Obesity (%)	6/30 (20.00%)	1/30 (3.33%)	0.103
Type 2 diabetes (%)	4/29 (13.79%)	0	0.112
Dyslipidemia	8/30 (26.67%)	3/31 (9.68%)	0.084
Hypertension (%)	8/30 (26.67%)	2/27 (7.41%)	0.083
Smoker (%)	11/28 (39.28%)	5/22 (22.73%)	0.213
Atherogenic risk (%)	8/30 (26.67%)	6/32 (18.75%)	0.456
Endothelial function			
Rest flow (PU)	11.28 ± 2.81	15.74 ± 5.88	0.017
Peak flow (PU)	51.36 ± 21.85	80.54 ± 29.11	0.006
Area of hyperemia (PU/s)	1730.93 ± 1398.64	2962.88 ± 1458.60	0.032
Peak flow − rest flow (PU)	39.27 ± 21.13	64.80 ± 25.25	0.008
Biological zero-peak flow (PU)	963.96 ± 584.07	1433.68 ± 565.25	0.040
Laboratory parameters			
HLA-B27 (%)	26/30 (86.67%)	…	
CRP ^a^, nmol/L	122.57 ± 171.90	13.62 ± 18.38	<0.001
ESR ^a^, mm/h	19.84 ± 22.42	7.48 ± 5.66	0.006
TNF-α, pg/mL	10.89 ± 10.00	4.02 ± 6.78	0.021
IL-1β, pg/mL	2.46 ± 1.20	1.57 ± 1.55	0.047
Cholesterol, nmol/L	4.90 ± 1.10	4.97 ± 0.71	0.800
HDL, nmol/L	1.09 ± 0.24	1.27 ± 0.30	0.014
LDL, nmol/L	3.26 ± 0.91	3.16 ± 0.66	0.619
Triglycerides, nmol/L	1.17 ± 0.60	1.14 ± 0.61	0.829
Apolipoprotein A, g/L	1.32 ± 0.25	1.42 ± 0.24	0.129
Apolipoprotein B, g/L	0.87 ± 0.21	0.85 ± 0.20	0.842
Treatments			
NSAIDs (%)	28/30 (93.33%)	…	
Sulfasalazine (%)	2/30 (6.67%)	…	

Values are presented as means ± SD. The data were analyzed using an independent samples *t*-test or a Mann–Whitney U test. ^a^ Non-normally distributed data. Differences in gender, obesity, type 2 diabetes, dyslipidemia, hypertension, smoking, and atherogenic risk distribution were analyzed by *χ*^2^ analysis or Fisher’s exact probability test. BASDAI, Bath Ankylosing Spondylitis Disease Activity Index; BASFI; Bath Ankylosing Spondylitis Functionality Index; BASMI, Bath Ankylosing Spondylitis Metrology Index; CRP, C-reactive protein; ESR, erythrocyte sedimentation rate; HDL, high-density lipoprotein; HLA, human leukocyte antigen; IL, interleukin; LDL, low-density lipoprotein, mSASSS, modified Stoke Ankylosing Spondylitis Spine Score; NSAIDs, nonsteroidal anti-inflammatory drugs; PU, perfusion units; PU/s, PU per second; TNF, tumor necrosis factor.

## Data Availability

All supporting data are provided in the current manuscript and the Supplementary Materials.

## Supplementary Materials

The following are available online at https://www.mdpi.com/article/10.3390/diagnostics11061037/s1: Table S1. Primer list; Table S2. Correlations between inflammatory, atherogenic, and oxidative mediators and endothelial function markers; Figure S1. Representative histograms of endothelial function in radiographic axial spondyloarthritis (r-axSpA) patients and healthy donors; Figure S2. Analysis of plasma atherogenic profile in radiographic axial spondyloarthritis (r-axSpA) patients.
