# Supplementary material for: Potential Role and Impact of Peripheral Blood Mononuclear Cells in Radiographic Axial Spondyloarthritis-Associated Endothelial Dysfunction
Source: Diagnostics (Basel). 2021 Jun 4;11(6):1037. doi: 10.3390/diagnostics11061037 (PMC8226914; doi:10.3390/diagnostics11061037)
Supplement: Supplementary file 1 [file diagnostics-11-01037-s001.zip › diagnostics-1201907-supplementary.pdf]

SUPPLEMENTARY MATERIALS

Figure S1

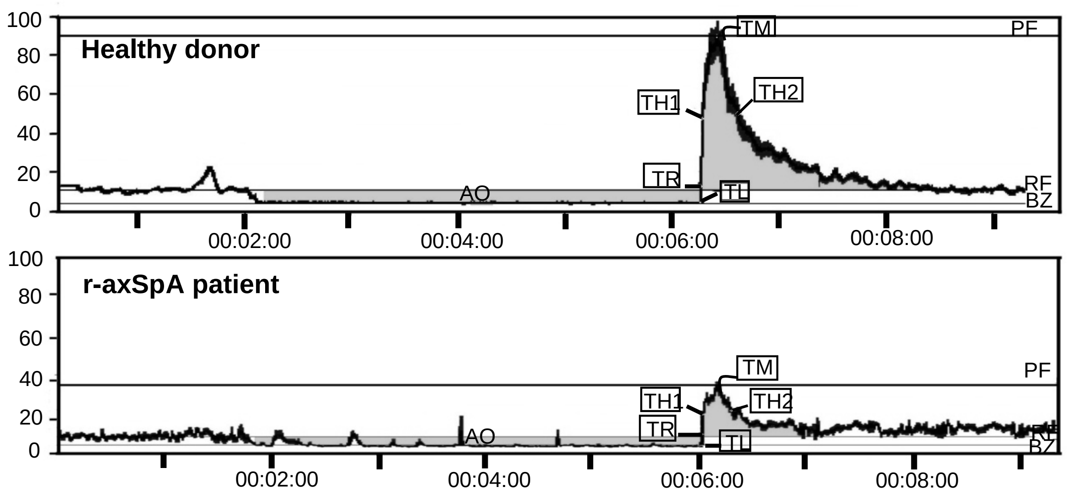

Figure S1. Representative histograms of endothelial function in radiographic axial spondyloarthritis (r-axSpA) patients and healthy donors. AO, occlusion area; BZ, biological zero; PF, peak flow; RF, Rest flow; TH1, time to half before hyperaemia; TH2, time to half after hyperaemia; TL, time to latency; TM, time to peak flow; TR, time to recovery.

Figure S2

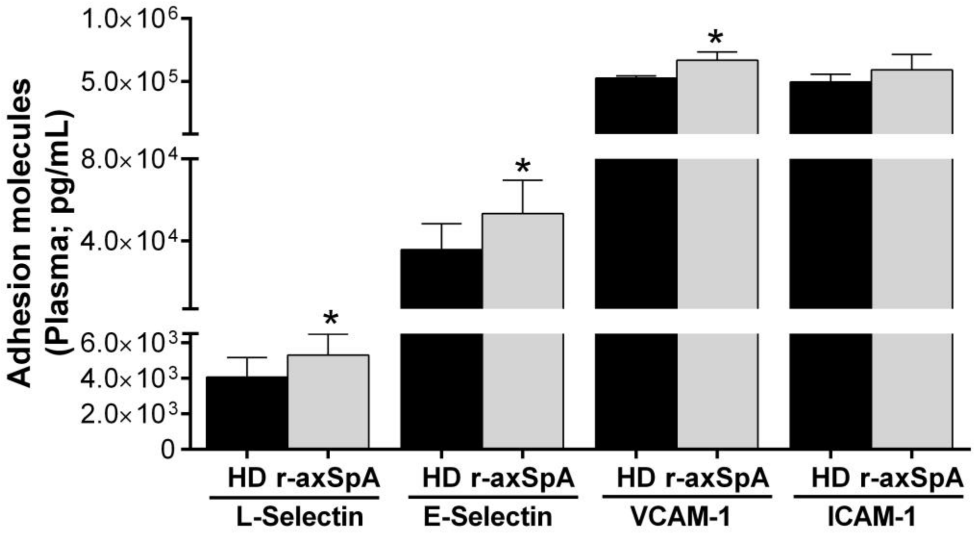

Figure S2. Analysis of plasma atherogenic profile in radiographic axial spondyloarthritis (r-axSpA) patients. Circulating levels of L-selectin, E-selectin, VCAM-1 and ICAM-1 by immunoassays. Values are presented as mean ± SD (HDs, n=32; r-axSpA patients, n=30). The data were analyzed using an Independent Samples *t* test. **vs*. HDs (*P*<0.05). HDs, healthy donors; ICAM, intercellular adhesion molecule; VCAM, vascular cell adhesion molecule.

Table S1. Primer list

Primer	Supplier	Sequence 5´-3´	
		Forward	Reverse
**GADPH**	Sigma-Adrich, St Louis, MI, USA	TGTAGTTGAGGTCAATGAAGGG	ACATCGCTCAGACACCATG
**ICAM-1**	Sigma-Adrich	GGGAACCAGAGCCAGGAGACACT	TGGGCCTCACACTTCACTGTCACC
**VCAM-1**	Sigma-Adrich	ACTTGATGTTCAAGGAAGAG	TCCAGTTGAACATATCAAGC
**THBS-4**	Sigma-Adrich	CAGGGTACGATTTTATGAAGG	TTCTGGGTTTGAAACTCTTG
**CDH5**	Sigma-Adrich	CGCAATAGACAAGGACATAAC	TATCGTGATTATCCGTGAGG
**L-selectin**	Sigma-Adrich	CAAGAGAAGTATGAATGACCC	TCAGGTAGAAATCTTCCCAG
**SOD1**	Sigma-Adrich	GAGCAGAAGGAAAGTAATGG	GATTAAAGTGAGGACCTGC
**SPP1**	Sigma-Adrich	GACCAAGGAAAACTCACTAC	CTGTTTAACTGGTATGGCAC
**TNF-α**	IDT, Leuven, Belgium	TCAGCTTGAGGGTTTGCTAC	TGCACTTTGGAGTGATCGG
**IL-1β**	Qiagen, Hiden, Germany	Not provided by the manufacturer	Not provided by the manufacturer
**IL-1α**	IDT	AGTTCTTAGTGCCGTGAGTTTC	GTGACTGCCCAAGATGAAGA
**IL-5**	IDT	TTGGCCCTCATTCTCACTG	GGAGAGTAAACCAATTCCTAGACT
**eNOS**	IDT	CTCATGGGCACGGTGATG	ACCACGTCATACTCATCCATACAC
**iNOS**	IDT	GCAGCTCAGCCTGTACT	CACCATCCTCTTTGCGACA
**TF**	IDT	TACTGTTTCAGTGTTCAAGCAGTGA	CAGTGCAATATAGCATTTGCAGTAGC
**STAT-3**	IDT	AGGCATTTGGCATCTGACAG	TGCTTCCCTGATTGTGACTG
**IL-2**	IDT	CTCCAGAGGTTTGAGTTTCCT	ACAAGAATCCCAAACTCACCA
**IL-6**	IDT	GCCCCACACAGACAGCCACTCACC	TGCCTCTTTGCTGCTTTCACACAT

**INF-γ**	IDT	CGACAGTTCAGCCATCACTT	GCAACAAAAAGAAACGAGATGAC
**IL-23**	IDT	GGCGCAGAGCCAGCCAGATT	ACCCTCAGGCTGCAGGAGTTGG
**CCL2**	IDT	GGGGAAAGCTAGGGGAAAATAAGT	CAGCAGCAAGTGTCCCAAAGAAG
**IL-10**	IDT	TCACTCATGGTCTTGTAGATGC	GCGCTGTCATCGATTTCTTC
**MIP-1α**	IDT	TGCTCGTCTCAAAGTAGTCAG	GCAACCAGTTCTCTGCATCA

Table S2

Table S2. Correlations between inflammatory, atherogenic and oxidative mediators, and endothelial function markers.

	**Rest Flow**	**Peak Flow**	**Area of hyperaemia**	**Peak Flow – Rest Flow**	**Biological Zero-Peak Flow**
**Lymphocytes**					
**STAT3**	**r = -0.431***	r = -0.174	r = -0.120	r = -0.143	r = -0.046
**IL-1α**	r = -0.215	**r = -0.541***	**r = -0.433***	**r = -0.521***	**r = -0.514***
**IL-1β**	r = -0.392	**r = -0.515***	r = -0.331	r = -0.437	r = -0.368
**TNF-α**	r = -0.333	**r = -0.475***	r = -0.346	**r = -0.438***	r = -0.368
**IL-23**	**r = -0.528***	r = -0.374	**r = -0.434***	r = -0.322	r = -0.203
**CDH5**	**r = -0.473***	**r = -0.442***	r = -0.305	r = -0.391	r = -0.259
**eNOS**	**r = -0.496***	r = -0.082	r = -0.174	r = -0.066	r = 0.032
**Monocytes**					
**STAT3**	r = -0.364	**r = -0.476***	**r = -0.402***	**r = -0.469***	**r = -0.477***
**SPP1**	r = -0.227	**r = -0.632****	**r = -0.562***	**r = -0.618***	**r = -0.497***
**Plasma**					
**TNF-α**	r = -0.288	**r= -0.463***	r = -0.196	**r = -0.482***	r = -0.323
**IL-1β**	r = -0.354	**r = -0.550***	r = -0.391	**r = -0.550***	**r = -0.508***
**E-selectin**	**r = -0.683***	r = -0.450	r = -0.400	r = -0.450	r = -0.167
**VCAM-1**	**r = -0.800***	r = -0.100	r = 0.200	r = -0.100	r = -0.100

r values of Spearman´s Rank correlation are shown. *$p<0.05$, **$p<0.01$.
